# A Perceptual Gap Analysis of Service Quality Perceptions in Home-Based Long-Term Care Service Centers

**DOI:** 10.3390/healthcare14080980

**Published:** 2026-04-08

**Authors:** Jui-Ying Hung

**Affiliations:** Department of Golden-Ager Industry Management, Chaoyang University of Technology, Taichung 413310, Taiwan; jybong@cyut.edu.tw

**Keywords:** home-based long-term care service centers, evaluatees, evaluators, service quality, two-dimensional gap analysis

## Abstract

**Background:** As Taiwan transitions into a super-aging society, the government has launched “Long-term Care (LTC) 3.0,” a policy initiative that marks a strategic shift from service expansion to integrated quality verification, digital oversight, and social resilience. This transition demands a robust quality verification mechanism. Ensuring perceptual consistency between service providers and external evaluators is critical for systemic fairness and sustainable service quality. **Objective:** This study utilized a two-dimensional gap analysis to examine the discrepancy in service quality benchmarks between home-based LTC center managers and assessment committee members, identifying critical divergence zones for institutional improvement. **Methods:** A cross-sectional evaluative study was conducted, involving center managers (evaluatees, n = 50) and external experts (evaluators, n = 28). The data were collected via a structured instrument covering 20 consensus benchmarks. **Results:** Significant perceptual gaps were identified across all dimensions (*p* < 0.001), with “Professional Care Quality” exhibiting the largest effect size (Cohen’s d > 1.5). Benchmarks with low external scores but high internal ratings were categorized into the “Overestimation (Management Blind Spot)” quadrant, signaling a systemic overestimation bias in administrative and clinical risk management. **Conclusions:** This study provides empirical evidence for the refinement of LTC 3.0 assessment systems. The results offer a strategic roadmap for policymakers to enhance organizational resilience by transitioning from subjective self-perception to objective, data-driven quality management through the two-dimensional gap model.

## 1. Introduction

### 1.1. Population Ageing Trends

As medical care improves, fertility and mortality rates decline, leading to rapid population aging. A super-aging society is reached in 2025 (people over 65 years old account for 21.0% of the total population) according to the National Development Council (NDC) and the Ministry of the Interior in Taiwan [1,2]. At the same time, the elderly support ratio—defined as the number of persons aged 65 and older that every 100 working-age persons (aged 15–64) must support—continues to rise, while the child support ratio declines. The accelerating aging of the population significantly increases the dependency burden on young adults.

### 1.2. The LTC Quality Framework

Because the social population structure is changing, the number of people in need of long-term care (LTC) is also increasing. As family care gradually weakens, the pressure for care on individuals and families has become increasingly intensified, which has in turn caused social and economic problems and impacted sustainable development. Therefore, establishing an integrated LTC system is a key issue in improving the social security system in Taiwan. The “LTC Services Act” classifies LTC centers into four main types based on service delivery: home-based, community-based, institutional residential, and integrated in Taiwan. Among them, home-based LTC centers and community-based LTC centers built the core framework for “aging in place” during the LTC 2.0 era. Crucially, the LTC 2.0 system significantly expanded eligibility beyond the elderly to include all individuals with physical or mental disabilities, regardless of age, persons with dementia aged 50 and over, and frail seniors. However, with the increase in demand for intensive care brought by a super-aging society, the policy focus of LTC 3.0 has shifted to strengthening institutional residential services and the “medical-care integration” capabilities of integrated institutions. This evolution from a “single service point” to a “one-stop integrated campus” reflects the LTC industry’s high emphasis on continuity of care. According to data at the end of 2024, home-based LTC centers account for 55.42% of all 4033 LTC centers, reflecting that in a super-aging society, elderly care still mainly meets the demand for “aging in place.” This is the important background for this study’s adoption of home-based LTC service centers (Table 1).

Despite the rapid quantitative expansion of home-based LTC centers under the LTC 2.0 framework, ensuring the consistency and reliability of service quality remains a formidable challenge for sustainable LTC management. While the Ministry of Health and Welfare (MOHW) has established a standardized “LTC Service Quality Verification Mechanism,” the current evaluation process often relies on top-down administrative compliance. As the demand for LTC service centers increases continually, the MOHW has implemented an LTC benefit and payment system since 2017 to provide case-centered care services [2]. According to the LTC 2.0 system, LTC services are subsidized for necessary items. After the disabled person is evaluated by the LTC service center, the payment is determined based on the approved disability level and the required service items. These service items include care management and policy encouragement, care and professional services, transportation, assistive devices, home barrier-free environment improvement, and respite services.

### 1.3. Challenges and Research Gaps

Article 1 of the LTC Services Act in Taiwan states the following: “To provide LTC for a regular LTC service system, ensure the quality of care and support services, and develop universal, diversified and affordable services, to protect the dignity and rights of service recipients and caregivers. Article 39 states the following: “The competent authority shall provide guidance, supervision, assessment, inspection and evaluation to LTC centers”. While Articles 1 and 39 of the LTC Services Act mandate the competent authorities to ensure service quality through supervision and evaluation, the rapid expansion of centers has exposed a critical gap in “Quality Planning” versus “Quality Control.” As institutions strive to meet standardized goals, the effectiveness of the assessment system is often hindered by a purely administrative approach to compliance.

Therefore, the purpose of the assessment of LTC service centers must effectively ensure that service quality helps the centers discover and understand their problems through the assessment system, and guides and supervises them through a self-improvement mode (internal and external control quality management mode) to meet the assessment standards (goals) and thereby improve service quality. Otherwise, centers must strengthen the management of institutional environmental facilities and equipment (hardware), professional case care capabilities, the integrity of the organization, and administrative operations (software) at all levels to protect the stakeholder benefits, improve the quality of LTC services, and protect the rights of LTC service users.

The quality improvement monitoring strategy has three aspects: quality planning, quality control, and quality improvement. “Quality planning” is to set standards and development indicators, “quality control” is to evaluate existing results: employees/customers meet the standards or expected results, and “quality improvement” is an important strategy. LTC center assessment refers to the systematic assessment and review of institutions that provide LTC services to ensure their service quality, operation management, and compliance with relevant laws and regulations. The purpose of the evaluation is to improve LTC services. According to the service level of the organization, the rights and interests of service recipients can be protected, and fair evaluation results can be provided for public reference. Therefore, the assessment of LTC centers has significance in improving service quality, protecting the rights and interests of service recipients, and promoting the healthy development of the LTC industry.

A significant research gap exists regarding the “Perceptual Alignment” between internal managers and external evaluators. According to empirical reports [3], several systemic tensions persist:

- Divergent Perceptions of Purpose: Centers often view assessment as a mere “data-gathering” burden unrelated to strategy, whereas the evaluation framework intends to drive “self-improvement.”
- Professional Knowledge vs. Practical Experience: A perceived “credibility gap” exists where managers question the practical expertise of committees, while committees criticize the lack of integrated management concepts within centers.
- Ambiguity of Benchmarks: Inconsistent interpretations of assessment standards lead to a “vague understanding” of the system’s purpose, often reducing it to a survival mechanism for securing government subsidies.

Unless a consensus on quality benchmarks is established between evaluatees and evaluators, the transformative potential of LTC assessment will remain unfulfilled. Understanding the cognitive consistency between these two stakeholder groups is essential for systemic fairness. Therefore, this study introduces a two-dimensional gap analysis framework to quantitatively examine the perceptual differences between 50 home-based LTC center managers (evaluatees) and 28 assessment committee members (evaluators) in central and southern Taiwan.

The primary objective of this study is to identify specific quality indicators with high perceptual divergence and provide evidence-based insights for optimizing the assessment system. By synchronizing the views of care providers (evaluatees) and evaluators, this study contributes to the literature by offering a diagnostic tool to enhance organizational effectiveness and systemic rigor in preparation for the upcoming LTC 3.0 (2026–2035) era in Taiwan.

## 2. Theoretical Review

### 2.1. LTC Centers

High-quality LTC services are increasingly critical as Taiwan enters a super-aging society. Since the promotion of the LTC 2.0 policy in 2017, the service model has shifted toward diversified and localized care to improve the quality of life for the elderly. High-quality care must be assessed through three interconnected pillars: structure, process, and outcome [1]. Recent international research reinforces this framework, suggesting that modern quality assessment must integrate technical excellence with functional aspects—such as patient-centered communication and responsiveness—to bridge the gap between service provision and recipient expectations [3]. Within the broader scope of healthcare systems, quality is increasingly viewed as a multidimensional construct that requires continuous monitoring of clinical safety, efficiency, and equitable access [3]. Current research on LTC service quality aligns with this triad, involving the assessment of service effects, management of providers, and satisfaction of recipients [2,4]. Despite continuous systemic improvements, the industry faces persistent challenges such as inconsistent quality standards and imperfect evaluation systems [5]. Internationally, these challenges are not unique to Taiwan. Otherwise, the evolution of the LTC insurance system highlights that maintaining standardized service quality across a rapidly expanding provider network is a systemic hurdle that requires rigorous external oversight in Japan [6].

To establish fair and transparent accreditation indices is essential. Previous studies have proposed evaluation indicators specifically for community and home-based LTC facilities to improve management efficiency and resource utilization [7,8]. However, a critical yet under-researched aspect of quality management is the perceptual alignment between internal and external stakeholders. In social care organizations, a significant gap often exists between how an institution evaluates its own service quality and how it is perceived by external assessment committees [9]. This gap is further complicated by the divergent priorities between service managers and recipients, as empirical evidence from primary health care suggests that internal providers often overestimate performance in areas that external users find less critical [10]. Understanding the viewpoints of both parties is essential for perfecting the evaluation system. To visualize and resolve these discrepancies, the Importance-Performance Analysis (IPA) and its derivative two-dimensional models have proven to be highly effective diagnostic tools in healthcare settings. Recent studies have underscored the scientific validity of the IPA tool in assessing hospital service quality by aligning it with WHO-defined dimensions such as safety, effectiveness, and patient-centeredness [11]. These models allow decision-makers to prioritize resource allocation by distinguishing between “critical quality attributes” and “over-fulfilled services,” taking targeted measures to improve service quality [2,12]. Ultimately, enhancing LTC services requires a multidimensional approach that integrates a complete evaluation index system with international evidence-based quality indicators [13,14].

### 2.2. Consensus Benchmarks and Methods

The cognitive alignment regarding assessment indicators and consensus benchmarks directly determines the functional integrity of the evaluation system. To ensure accurate service leveling and continuous improvement, LTC centers must not only understand but also effectively internalize these indicators. The assessment framework is strategically designed across three dimensions: consensus benchmark development and definition, application proficiency, and organizational training. This multidimensional approach aligns with international social governance models, which emphasize that sustainable development in elderly services requires a synergy between standardized policy frameworks and the active engagement of diverse stakeholders [15]. In Taiwan, the MOHW mandates the annual announcement and adjustment of assessment indicators, which are then administered by local LTC offices. Recent qualitative evidence from international urban healthcare settings highlights that the successful implementation of such indicators often faces systemic barriers, including administrative burdens and professional burnout, while facilitators such as digitized record-keeping and robust institutional support are critical for maintaining service quality [16]. For home-based LTC centers, the evaluation core comprises three primary concepts: Management Effectiveness, Professional Care Quality, and Individual Equity Guarantee. These concepts are operationalized through 20 consensus benchmarks, measured on a five-point scale (totaling 100 points) (Table 2).

The results of this assessment carry significant administrative weight: a score of 70 or above is deemed “qualified,” allowing centers to renew their service contracts, whereas a score below 70 results in “unqualified” status and the immediate suspension of care services. Such high-stakes outcomes underscore the necessity for precise cognitive synchronization between internal managers and external committees. If a “perceptual gap” exists—where managers’ self-assessments significantly diverge from committee evaluations—it can lead to systemic failures in identifying actual service deficiencies. Therefore, analyzing these 20 benchmarks through a two-dimensional gap analysis is essential for validating the reliability of the current assessment regime.

The participants of this study comprised two primary stakeholder groups: internal business managers (evaluatees) and external assessment committee members (evaluators) of home-based LTC centers.

- Evaluatees (Internal Perspective): As the primary leaders responsible for institutional operations and strategic management, these managers possess an in-depth, localized understanding of their organizations. Their insights provide an essential “bottom-up” view of how the evaluation system is implemented in daily practice.
- Evaluators (External Perspective): These members are responsible for the independent and objective supervision of LTC centers. Leveraging their multi-institutional experience and professional expertise, they provide a “top-down” standardized assessment.

By analyzing the perceptions of these two distinct groups, this study captures the dual perspective necessary to identify the “perceptual gaps” (as defined in our two-dimensional gap analysis) that may hinder the effectiveness of the national LTC quality assurance framework.

## 3. Materials and Methods

### 3.1. Study Design and Data Collection

This research employs a quantitative, cross-sectional comparative design utilizing official on-site assessment records collected between 2023 and 2025 from home-based LTC service centers in Taiwan. The study population comprises 50 service providers (evaluatees) and 28 external committee members (evaluators) who participated in the mandatory annual accreditation program. The data were retrieved directly from the official government evaluation database, ensuring that the analyzed scores reflect formal, standardized assessment results rather than retrospective self-reports.

To ensure high external validity, this study employed a census-based approach (purposive population-wide sampling) within the target county:

- Selection Criteria: All 57 home-based LTC centers currently registered and active in the target county were screened. Exclusion criteria were applied to 7 newly established centers (with less than one year of operation) as they were not yet subject to statutory assessment during the 2023–2025 cycle.
- Sample Characteristics: Of the 50 eligible centers, all 50 provided valid assessment data (100% participation rate).
- Data collection: followed a rigorous, two-stage administrative procedure within the official 2023–2025 accreditation cycle.
  (1) Stage 1: Internal Self-Assessment (*Y*-axis data). Before the external audit, each center manager was required to perform a self-evaluation using the official “Home-based LTC Service Quality Assessment Scale.” This was completed via a secure government web portal, representing the institution’s internal perception of its service performance.
  (2) Stage 2: External Expert Assessment (*X*-axis data). Subsequently, a committee of 28 experts (assigned by the MOHW or local health bureaus) conducted on-site inspections. Each audit team typically consisted of three members (majors are: Administration, Social work, and LTC Nursing). After reviewing physical evidence and conducting interviews, the committee reached a consensus score for each of the 20 benchmarks.

This dual-source, multi-stakeholder approach allows for a rigorous comparison between internal institutional perceptions and objective external benchmarks. Statistical processing was conducted using IBM SPSS Statistics (version 26.0; IBM Corp., Armonk, NY, USA), employing descriptive statistics to summarize institutional characteristics and a modified two-dimensional gap analysis to identify perceptual divergences across the 20 assessment benchmarks.

### 3.2. Methodological Framework: Two-Dimensional Gap Analysis

Importance–Performance Analysis (IPA), originally proposed by Martilla and James (1977) [18], is a widely recognized method for measuring the relative importance of service attributes against actual performance. Although the classical model utilizes an “Importance-Performance” dyad, this study adopts a modified two-dimensional IPA framework to conduct a Perception Gap Analysis.

Instead of the traditional axes, this research utilizes the IPA matrix structure to map “Evaluator Performance (External)” on the *X*-axis against “Evaluatee Performance (Internal)” on the *Y*-axis. This adaptation follows the evolving diagnostic logic of IPA in service quality research, where the matrix serves as a strategic tool to identify Institutional Calibration discrepancies between different stakeholders [11,19].

By substituting the axes, the model effectively categorizes service indicators into four diagnostic quadrants:

- Consensus (High/Low Performance): Where internal and external views align.
- Blind Spots: Where internal self-assessment significantly exceeds external verification (comparable to the “Concentrate Here” priority in the classical model).
- Hidden Strengths: Where external evaluators identify performance that the institution may be underestimating.

This modified approach provides a more granular understanding of quality management gaps in the LTC context, moving beyond mere satisfaction to identify systemic perceptual divergences [20] (Table 3).

### 3.3. Data Analysis and Scoring Standards

The data analysis was supported by a specialized assessment software developed under the 2023–2025 LTC Service Quality Improvement Plan [4]. This tool utilizes a non-linear five-grade scale (A+, A, B+, B, C) mapped to numerical values (5, 4.25, 3.5, 1.75, 0), which is the standardized scoring system mandated for official LTC accreditation in Taiwan.

The value of 3.5 for Grade B+ is specifically designated as the “Compliance Threshold” (representing 70% of the total score), which aligns with the official passing requirements. The non-linear nature of this scale—specifically the weighted drop between B+ (3.5) and B (1.75)—is strategically employed by regulators to emphasize the critical difference between “Meeting Standards” and “Failure to Comply.” Utilizing this official weighting ensures that the performance gaps identified in our modified IPA model are policy-relevant, highlighting areas where institutions are at risk of failing national quality audits.

To identify significant perceptual gaps, a specific “divergence threshold” was established: a discrepancy was flagged if a committee member’s score fell below the 3.5 threshold, or if there was a ≥2-grade difference compared to the center’s self-assessment. These flagged cases underwent further qualitative review to identify the root causes of the divergence and facilitate targeted improvements.

To ensure the rigor and validity of the findings, data analysis was conducted in several sequential stages.

- First, the reliability and structural validity of the assessment instrument were verified. Internal consistency was evaluated using Cronbach’s α, with a coefficient exceeding 0.70 considered indicative of acceptable reliability.
- Prior to performing comparative analyses, assumption testing was conducted to ensure the appropriateness of parametric procedures. The data were subjected to the Kolmogorov–Smirnov test to assess normality, while Levene’s test was employed to evaluate the homogeneity of variance. In instances where the assumption of equal variances was violated (*p* < 0.05 in Levene’s test), Welch’s *t*-test was utilized to provide a more robust and accurate comparison of mean scores.
- To identify perceptual divergences between the two primary groups, Independent Samples *t*-tests (or Welch’s *t*-test where applicable) were performed to compare the mean scores of service providers (evaluatees) against those of the committee members (evaluators) across all indicators and dimensions.
- Furthermore, a Gap Analysis was conducted to quantify the discrepancy (defined as the difference between evaluatees’ and evaluators’ scores). Statistical significance for all tests was set at a threshold of *p* < 0.05. Finally, to determine the magnitude of the observed differences, Cohen’s d was calculated as the effect size, providing a standardized measure of the perceptual gap between the two cohorts.

## 4. Results and Discussion

### 4.1. Descriptive Statistics of Background Variables of the LTC Centers

The summary of home-based LTC centers (n = 50) and their service recipients (n = 7166) provides a critical baseline for interpreting the subsequent perception gap analysis (Table 4).

- Organizational Maturity and Expansion: Over half of the surveyed centers (56.0%) have been established for less than three years. This indicates that the sector in the target county is in a rapid expansion phase, where newer organizations may still be refining their internal quality management systems relative to established regulatory standards.
- Service Intensity and Clinical Complexity: Although 62.0% of the centers are small-to-medium-sized (serving fewer than 100 cases), the clinical complexity of the caseload is high. Specifically, 55.35% of service recipients are classified as having moderate-to-severe disability (CMS Levels 4–8). Such a high concentration of severe cases necessitates rigorous institutional risk management. This context explains the substantial pressure on Concept B (Professional Care Quality) and provides a logical foundation for the discrepancies observed between internal self-assessments and external expert evaluations.

**Table 4:** Descriptive statistics of background variables of the on-site LTC centers.

Variable	Category	n (%)
Age of the Center	<3 years	28 (56.0%)
	3–7 years	11 (22.0%)
	>7 years	11 (22.0%)
Classification ^1^	Public/LTC Foundation	4 (8.0%)
	Individually Established	22 (44.0%)
	Foundation/Association	14 (28.0%)
	Affiliated Organization	10 (20.0%)
Cases Served	<100 cases	31 (62.0%)
	100–200 cases	10 (20.0%)
	>200 cases	9 (18.0%)
Disability Level ^2^	Mild (Level 2–3)	3192 (44.54%)
	Moderate (Level 4–6)	2827 (39.45%)
	Severe (Level 7–8)	1139 (15.90%)

^1^ Classification: Includes public entities, specialized LTC foundations/corporations, and general non-profit organizations providing affiliated LTC services. ^2^ Disability Levels: Based on the MOHW’s Care Management System (CMS). Mild refers to individuals requiring assistance primarily with instrumental activities of daily living (IADLs); Moderate involves significant dependence for physical activities of daily living (ADLs); Severe indicates near-total dependence, often requiring 24 h monitoring or specialized medical care.

### 4.2. Cognitive Differences Between Evaluatees and Evaluators

As illustrated in Table 5, the discrepancy between evaluatees (n = 50, M = 4.59) and evaluators (n = 28, M = 3.99) was statistically significant across all dimensions (*p* < 0.001). Evaluatees consistently reported significantly higher perceptions compared to evaluators. Notably, the concept of Professional Care Quality exhibited the most substantial gap (0.735), followed by the concept of Management Effectiveness (0.603). To assess the practical significance of these differences, Cohen’s d was calculated; all effect sizes exceeded the ‘large’ threshold (d > 1.3), confirming that the perception gap is a robust systemic phenomenon rather than a marginal variance. The 95% confidence intervals further indicate that while evaluatees perceive their service quality at a near-optimal level (>4.5), external evaluators identify significant room for improvement, particularly in professional care standards.

The comparative analysis between self-assessment scores (evaluatees) and committee assessment scores (evaluators) reveals significant perceptual gaps across the majority of the consensus benchmarks. As shown in Table 6, independent samples *t*-tests indicate that service providers consistently assigned higher scores to their own performance compared to the external committee’s ratings (*p* < 0.001).

The item-level analysis (Table 6) identifies a pervasive ‘Leniency Effect’ among evaluatees, with 19 out of 20 benchmarks showing statistically significant overestimation (*p* < 0.001). The most critical discrepancies were observed in ‘A8: Regular health check-ups and follow-ups for staff’ (M.D. = 1.215, d = 1.54) and ‘B5: Accident and emergency handling and prevention’ (M.D. = 1.035, d = 1.61). These ‘Extreme Gaps’ indicate that while LTC center managers perceive their internal protocols as near-optimal, external evaluators identify substantial operational risks. In contrast, ‘A5: Information system coding’ emerged as the only benchmark of consensus (*p* = 0.109, d = 0.38), suggesting that quantifiable administrative tasks are more easily aligned than complex care-related competencies.

### 4.3. Two-Dimensional Gap Analysis

**Figure 1:**
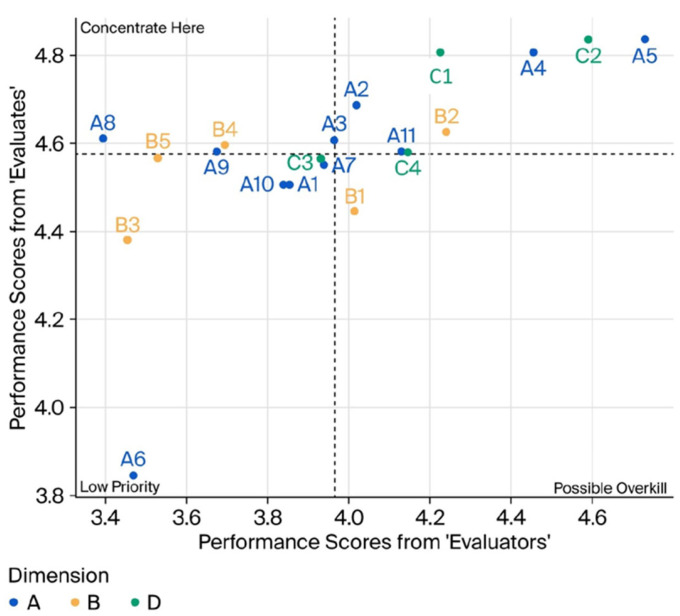
Two-Dimensional Gap Matrix for Perceptual Gap Analysis.

This study adopts a modified two-dimensional gap matrix to conduct a robust perception gap analysis. In this adapted model, the *X*-axis represents the performance scores provided by ‘Evaluators’ (External Committee Evaluation, M = 3.987), while the *Y*-axis reflects the scores from ‘Evaluatees’ (Internal Self-Assessment, M = 4.591). The matrix serves as a diagnostic tool to identify perceptual discrepancies between diverse stakeholders. By substituting the traditional axes with internal and external perspectives, the model effectively categorizes 20 consensus benchmarks into four distinct quadrants. This classification allows for the identification of consensus areas, management blind spots, and hidden strengths, thereby providing a more granular and actionable understanding of quality management gaps within the LTC context (Figure 1).

Using the recommendations, centers can prioritize the indicators that need improvement and those with advantages. The results can be used to improve the overall service quality, enhance the protection of the rights and interests of service recipients, and improve the center’s operational efficiency and the credibility of external evaluations.

The analysis results of the indicators showed that the performance and importance of centers in different quadrants were different. The consensus benchmark in the four quadrants is shown in Table 7.

Quadrant I ⌈Consensus Excellence⌋

The Quadrant, including A2 (work manuals), A4 (financial management), and A5 (information system coding), represents the “Consensus Excellence” of the centers. These benchmarks reflect a high degree of alignment between internal operational standards and external regulatory expectations. Notably, A5 achieved a state of statistical congruence (*p* = 0.109), suggesting that the technical infrastructure and administrative protocols of these centers have matured into a standardized, reliable system. From a strategic perspective, these areas are categorized under “Stable Development,” requiring continuous quality maintenance rather than drastic reform. The management should leverage these established Structural Indicators as the baseline for organizational scaling. By institutionalizing these successful SOPs (Standard Operating Procedures), centers can ensure long-term sustainability and compliance. Furthermore, these consensus strengths should serve as the “stabilizers” for the organization, providing the necessary resource buffer to reallocate focus toward addressing the critical gaps identified in other quadrants.

Quadrant II ⌈Critical Improvement/Overestimation⌋

Indicators located in Quadrant II, such as A8 (staff health follow-ups), A9 (pre-training), and B4 (case management), represent critical “Management Blind Spots” where internal managers perceive near-optimal performance (M > 4.5) despite significantly lower ratings from evaluators (M < 4.0). This perceptual discrepancy reveals a systemic overconfidence bias, likely stemming from a reliance on traditional, manual record-keeping that fails to trigger alerts for missed deadlines or non-compliance during frequent personnel turnover. From a strategic perspective, these centers must transition from passive documentation to active digital early-warning systems. Implementing AI-integrated tracking for staff professional development and automated case-status monitoring is essential to bridge this gap. By rectifying these blind spots, LTC centers can ensure that administrative safeguards are not merely perceived as completed but are rigorously executed to meet professional standards, thereby protecting both staff welfare and service recipient rights.

Quadrant III ⌈Consensus Weakness⌋

The Quadrant III, including A1 (business plan implementation), B3 (cross-professional referral), and B5 (accident and emergency prevention), is classified as “Consensus Weaknesses.” Unlike the blind spots in Quadrant II, both evaluatees and evaluators assigned significantly lower scores (M < 4.0 for evaluators), reflecting a mutual recognition of systemic deficiencies. From a strategic improvement perspective, this quadrant represents the “Low Resistance Reform Zone.” Because the internal perception of these functions is not inflated, management can more easily mobilize organizational resources for change. Specifically, for B5, centers should move beyond theoretical protocols to active organizational drills and risk-control simulations to enhance real-world crisis awareness. Furthermore, the weakness in B3 underscores the need for a shift toward Interdisciplinary Collaboration. By fostering external partnerships and formalizing referral pathways, centers can bridge the gap between basic care and comprehensive service networks. Addressing these shared deficiencies is vital for evolving from a “compliance-based” facility to a high-quality, resilient care provider in the competitive LTC market.

Quadrant IV ⌈Potential Advantage/Underestimation⌋

Indicators situated in Quadrant IV, specifically B1 (professional knowledge and skill enhancement), represent the “Hidden Strengths” of the centers. While internal evaluatees expressed a lower self-perception (M = 4.45) relative to other dimensions, external evaluators assigned significantly higher scores (M = 4.02, *p* < 0.001), identifying a robust foundation in continuing education. This “Internal Modesty” or lack of self-confidence suggests that while LTC personnel have successfully integrated professional growth into their daily practice, the institution may fail to recognize or leverage this competence as a competitive advantage. From a strategic management perspective, centers should transition from a “compliance-oriented” training model to a “Professional Branding” strategy. By formalizing the recognition of staff expertise and encouraging peer-led clinical sharing, institutions can transform these latent competencies into visible quality markers. Leveraging these unrecognized assets not only boosts staff morale but also enhances the institution’s external reputation, positioning it as a leader in professional care standards within the super-aging society.

## 5. Discussion

### 5.1. The Divergence in Quality Logic: Objectivity vs. Subjectivity

The significant perception gap identified in Management Effectiveness underscores a fundamental conflict between “Managerial Logic” and “Evaluative Logic.” While center managers focus on the existence of administrative systems (Structural Quality), the assessment committee prioritizes the objectivity of outcomes (Outcome Quality), reflecting the classic Donabedian triad [1].

This “Managerial Overconfidence” is consistent with the Social Desirability Bias observed in previous studies, where internal stakeholders tend to overestimate performance to maintain organizational morale [9,10]. Specifically, our findings mirror the empirical evidence from primary health care, where internal providers often overestimate performance in areas that external users or evaluators find less critical [10]. This suggests that self-assessment in Taiwan’s LTC centers currently lacks the data-driven precision required to match external professional benchmarks [21], a challenge also noted in Japan’s LTC insurance system, where maintaining standardized quality across expanding networks requires rigorous external oversight [6].

### 5.2. Organizational Resilience: Stability vs. Emergency Capacity

Regarding Professional Care Quality, a distinct mismatch in quality focus was identified. Managers prioritize the “Stability of Daily Operations,” whereas evaluators emphasize “Organizational Resilience.” This discrepancy highlights a potential “Institutional Blindness,” where long-term immersion in routine care desensitizes internal staff to high-risk contingencies, such as emergency handling (B5).

This aligns with Safety Culture theory and recent international literature suggesting that modern quality assessment must move beyond routine technical excellence to include functional responsiveness [3]. The “Blind Spots” identified in our IPA matrix—where external ratings are significantly lower than self-assessments—echo the systemic barriers found in other urban healthcare settings, such as administrative burdens overshadowing risk-prevention capacity [16]. Our results suggest that for LTC 3.0 transition to succeed in Taiwan, the focus must shift from “maintaining status quo” to “enhancing resilience,” consistent with the WHO-defined dimensions of safety and effectiveness [11].

### 5.3. Implementation Efficacy in Individual Equity

The discrepancy identified in Individual Equity Guarantee reflects a critical gap between “System Integrity” and “Implementation Efficacy.” While managers focus on the formal completion of legal contracts and administrative compliance, evaluators demand functional responsiveness in grievance processing and equity protection.

This suggests that LTC centers may suffer from a “Formalism Trap,” where the mere presence of a feedback system is mistaken for its effective operation. This finding aligns with the multidimensional construct of quality [3], which emphasizes that technical excellence must be integrated with functional aspects—such as responsiveness—to bridge the gap between service provision and recipient expectations.

Furthermore, our results support the international social governance models [15], which argue that sustainable development in elderly services requires a synergy between standardized policy frameworks and the active engagement of stakeholders. In the absence of automated tracking or digitized record-keeping—tools identified as critical facilitators in international healthcare settings [16]—managers often overlook the actual experience of service recipients. This leads to the identified subjective bias, where “procedural justice” (on paper) fails to translate into “perceived equity” (in practice).

### 5.4. Strategic Roadmap via Two-Dimension Gap Matrix

By repurposing the two-dimensional gap Matrix framework as a diagnostic tool, this study provides a specialized roadmap for institutional excellence.

#### 5.4.1. Addressing Management Blind Spots (Quadrant II: Prioritize Improvement)

Indicators in Quadrant II, such as A8 (staff health follow-ups) and B4 (case management), represent the most urgent “Management Blind Spots.” Although the current quantitative data does not directly identify causal mechanisms, the robust statistical gaps (*p* < 0.001, d > 1.5) suggest plausible hypotheses related to institutional constraints. The observed overconfidence may stem from a reliance on manual record-keeping, which lacks the automated triggers necessary to sustain compliance during frequent personnel turnover. Future research using qualitative interviews is required to validate these exploratory interpretations and confirm whether digital early-warning systems can effectively close these critical gaps.

#### 5.4.2. Leveraging Hidden Strengths (Quadrant IV: Potential Advantage)

Quadrant IV reveals “Hidden Strengths,” particularly in B1 (professional knowledge enhancement). The fact that external validation exceeds internal perception suggests a level of “Internal Modesty” unique to the Taiwanese LTC context. Strategically, institutions should transition from a compliance-oriented training model to a “Professional Branding” approach, formalizing the recognition of staff expertise to transform these latent competencies into visible market advantages.

#### 5.4.3. Consensus-Based Management (Quadrants I & III)

Quadrant I reflects “Consensus Excellence” (e.g., A5 information system coding, *p* = 0.109), where matured Structural Indicators serve as organizational stabilizers. Conversely, Quadrant III identifies “Consensus Weaknesses” (e.g., B3 cross-professional referral). As both parties recognize these deficiencies, this quadrant serves as a “Low Resistance Reform Zone.” Management should capitalize on this mutual awareness to mobilize resources for interdisciplinary collaboration, evolving from basic compliance to a resilient, high-quality service model.

### 5.5. Strategic Suggestions and Research Limitations

#### 5.5.1. Practical Suggestions for Policy and Management

To improve the accuracy and consistency of quality assessment, the following strategies are proposed:

- Refining Assessment Standards and Guidance: The level of understanding regarding consensus benchmarks directly affects self-assessment accuracy. Specific assessment standards must be formulated in advance with detailed instructional guidance. By providing concrete examples of practical applications, managers can better grasp the nuances of each indicator, improving the reliability of internal evaluations.
- Dynamic Weighting and Hierarchical Assessment: The weighting of benchmarks should be adjusted to avoid the undue impact of a single item on the overall score. By conducting a hierarchical assessment based on the relative importance and influence of each indicator, a more nuanced evaluation of institutional performance can be achieved, allowing for the identification of specific problems across different service domains.
- Establishing Continuous Tracking and Coaching: Quality assessment should transition from a “one-time inspection” to a continuous improvement cycle. A problem follow-up and coaching plan should be established to track shortcomings discovered during evaluation. This feedback loop helps organizations integrate quality improvement into daily operations rather than merely preparing for periodic audits.
- Balancing Standardization with Flexibility: The assessment system for home-based LTC centers must balance rigorous standardization with operational flexibility. By introducing flexible standards and resource support for institutions with limited capacity, the policy can promote sustainable development and ensure the long-term fairness and transparency of the LTC service system.

#### 5.5.2. Methodological and Contextual Limitations

Despite the diagnostic insights provided by the modified IPA matrix, several critical limitations must be acknowledged to contextualize the findings:

- Sample Scope and Generalizability: This study was conducted within a single geographical region in Taiwan, encompassing 50 home-based LTC centers. While these centers represent a significant portion of the local service network, the limited sample size and regional homogeneity may constrain the generalizability (external validity) of the results to other regions with different socio-economic profiles or urban-rural resource disparities. Future research should aim for a nationwide analysis to validate whether these perception gaps persist across broader geographic contexts.
- Systemic Measurement Bias: The reliance on data derived from an official governmental evaluation system introduces potential institutional bias. In Taiwan’s “high-stakes” audit environment, where scores are directly tied to institutional contracting and subsidies, a “Performance-Driven Response Bias” is likely. Managers may instinctively inflate self-assessment scores as a defensive organizational strategy, while committee scores—though professionally audited—provide only a cross-sectional snapshot during pre-scheduled visits. This systemic “Audit Culture” may inherently amplify the discrepancies between internal and external perceptions.
- Lack of Stakeholder Triangulation: This study primarily focuses on the dyadic relationship between managers and expert evaluators. The exclusion of direct feedback from service recipients and their families (User-Perceived Quality) means that the “true” service excellence remains partially obscured. Future investigations should utilize Data Triangulation, integrating recipient satisfaction surveys with longitudinal digital operational tracking to provide a more holistic verification of care quality.

## 6. Conclusions

### 6.1. Principal Findings and Theoretical Insights

The core findings reveal systemic “Management Blind Spots” (Quadrant II) in administrative follow-ups and “Hidden Strengths” (Quadrant IV) in professional knowledge enhancement. These statistical discrepancies underscore a fundamental tension between Managerial Logic—which often relies on subjective, manual oversight—and Evaluative Logic, which demands objective, data-driven compliance. The identified gaps provide empirical evidence that institutional quality in LTC is not a static benchmark but a negotiated perception that requires continuous alignment between stakeholders.

### 6.2. Scientific and Academic Contributions

The scientific significance of this research extends beyond its regional context, offering three distinct contributions to the broader academic discourse on healthcare management:

- Deconstructing Systematic Discrepancies: This study provides robust empirical evidence of a systematic divergence between institutional self-evaluation and external professional auditing. By quantifying a pervasive “Leniency Effect” across 19 of 20 benchmarks, we contribute to Organizational Behavior literature, suggesting that in “high-stakes” regulatory environments, self-assessment often functions as a defensive administrative ritual rather than a reflective quality tool.
- Methodological Advancement in Perception Alignment: We demonstrate that quality in the LTC sector is a multidimensional, stakeholder-dependent construct. By repurposing the IPA framework into a two-dimensional perception gap matrix, this research offers a novel diagnostic mechanism to identify where internal “Managerial Logic” fails to align with external “Evaluative Logic.” This provides a visual roadmap for Institutional Calibration that transcends simple mean-difference testing.
- Contribution to Evaluation Theory: Our findings highlight the inherent limitations of subjective, checklist-based audit systems. By revealing specific “Management Blind Spots” in emergency resilience and staff follow-ups, this study argues for a paradigm shift in Evaluation Methodology—moving from static, one-time inspections toward a “Continuous Calibration Model” that integrates digital evidence with stakeholder triangulation.

### 6.3. Practical and Policy Implications

The practical contributions emphasize the urgent need for LTC centers to transition from passive documentation to active digital early-warning systems. Closing these perception gaps is not merely an administrative requirement but a strategic necessity for enhancing organizational resilience and ensuring equity for service recipients in a super-aging society. To ensure that policy and organizational improvements are directly grounded in the study’s empirical findings, the following recommendations are structured based on the identified perception gaps (Table 8).

Ultimately, achieving excellence in home-based care requires balancing internal operational flexibility with external professional standards. This study serves as a critical baseline for policy-makers and center managers to foster a more transparent, evidence-based culture of quality in the evolving LTC 3.0 landscape.

## Figures and Tables

**Table 1:** Classification and Roles of LTC Service Centers in Taiwan (End of 2024).

Center Type	Service Characteristics	Establishments in Taiwan (End of 2024)	Key Points in LTC 3.0
Home-based	Provides physical care, daily living support, and medical nursing within the recipient’s home.	2235 lefts (55.42% of total LTC lefts)	Digitalized Precision Dispatch & Smart Monitoring: Utilizing AI to optimize matching efficiency and employing wearable devices for in-home risk pre-warning.
Community-based	Provides Day Care and Family Care, emphasizing social interaction and delaying functional decline.	1478 lefts (Including Day Care, Family Care, Group Homes, and Small-scale Multi-function) (36.65% of total LTC lefts)	AI-powered Precision Exercise & Disability Prevention: Implementing Generative AI and digital rehabilitation tools to provide personalized health promotion programs.
Residential-based	Provides 24 h full-time care, targeting individuals with moderate-to-severe disability or dementia.	122 lefts (Including Senior Welfare Institutions and General Nursing Homes) (3.03% of total LTC lefts)	Medical-Care Integration & Increased Subsidies: Serving as the backbone for heavy-care needs, strengthening “green channels” with hospitals, and significantly increasing resident subsidies.
Integrated	A single legal entity providing two or more types of services (e.g., Day Care + Home-based Care).	198 lefts (4.91% of total LTC lefts)	One-stop Smart Care Campus: Acting as a regional resource integration hub to implement “Continuity of Care” and reduce service fragmentation for the elderly.

**Table 2:** Assessment Consensus Benchmark for home-based LTC service centers.

Assessment Concept	Consensus Benchmark	
	Home-Based LTC Centers	Key Content of Benchmark
A: Management effectiveness	A1: Business plan development and implementation	Constructing the annual business plan based on the mission and goals of the organization’s development. Reviewing and revising the plan and keeping records regularly. Propose review and improvement strategies based on the achievement of planned goals.
	A2: Work manuals and administrative management regulations	Establish a work manual and regularly revise management regulations. The manual should include the organizational structure of the center, the responsibilities of personnel, key work processes, procedures for handling emergencies or incidents, infection control and infectious disease prevention measures, and staff self-health management guidelines.
	A3: Supervisory system operation	Hold administrative meetings regularly (at least quarterly) to discuss service quality and work improvement.
	A4: Financial management system	Establish an independent accounting system based on the principle of accrual accounting with tax reporting information.
	A5: Information system coding	Service providers must record the service status of each case in the municipal government care service management information platform by the 10th of the following month.
	A6: Deficiency and improvement evaluation by the relevant authority during the auditing/inspection period	During the assessment period, any suggested improvements received from supervisory authorities in inspections/audits or guidance/supervision (including fire and labor authorities) can be concretely implemented and tracked. A+, as improvement reached 100% A, as improved by 80% but did not reach 100%. B+, as improvement reached 60% but did not reach 80% B, as improvement reached 40% but did not reach 60% C, as improvement did not reach 40%
	A7: Establishment and implementation of systems related to staff rights and interests	Establish systems related to staff rights and benefits, including: employment (appointment), salary (labor compensation), welfare (such as accident insurance, caregiver responsibility insurance, etc.), reward mechanisms, attendance, retirement and pension systems, performance evaluation and disciplinary measures, education and training, grievance procedures, human resource development systems, and personal safety mechanisms. Annual retention rate.
	A8: Regular health check-ups and follow-ups for staff	LTC staff undergo health check-ups each year (within 365 days). The examination items might include: chest X-ray, routine blood and biochemical tests, and urinalysis, with complete records maintained. Vaccination status of staff. Follow up on individual examination results and conduct case management.
	A9: Pre-training for new staff	New staff should receive at least 8 h of pre-employment training within 3 months; this training includes: an introduction to the overall environment, explanation of the work manual, occupational health and safety education, infection control, emergency response procedures, and hands-on practice of service items. Pre-employment training should include effectiveness evaluations, encompassing organizational competency assessments and surveys or feedback forms from the evaluated personnel.
	A10: Actual participation of supervisor in administrative and care quality management meetings/activities	The business manager is actively involved in administration and care quality management, and keeps records (such as constructing the annual business plan, presiding over administrative meetings, reviewing meetings for accidents or emergency incidents, etc).
	A11: Establishment and implementation of a scheduling mechanism for caregiving staff	Establish a reasonable assignment or scheduling module for care service workers. Rationality of case assignment or scheduling: service provision times can be determined based on the care needs and difficulty of care for each service recipient.
B: Professional care quality	B1: Strengthening the professional skills of LTC service personnel	Each LTC staff member must participate in continuing education or accumulate at least 20 h each year. Each LTC staff member should complete 20 h of training courses on dementia care services and possess a certificate of completion.
	B2: Caregiver service execution and feedback from service recipients/family members	Caregivers carry out the services according to the service plan and keep records. Caregivers can respond to the needs of the service recipients promptly, and interviews with the service recipients/families indicate positive feedback regarding the received services.
	B3: Proactive referral of cross-professional services	According to the care needs of the service recipients at different stages, timely referral to medical or other professional services, such as home medical care, palliative home care, and home rehabilitation. Hold at least one interdisciplinary case discussion meeting quarterly and keep a record. Interprofessional case discussion meetings must include at least 2 different professional fields.
	B4: Management of case starting and finishing for service recipients	Formulate procedures and processing guidelines for case initiation/acceptance, referral, service suspension, and case closure, and clearly explain them to service recipients and their families.
	B5: Accident and emergency handling and prevention	Establish emergency or incident handling procedures and processes that align with the characteristics of the service. Execute and record according to the handling procedure when it occurs. Make analytical reports on the events that occurred, review and improvement measures, and follow-up records.
C: Individual equity guarantee	C1: Signing of a service contract with the service recipient or family member	Sign a contract with the client (the person themselves, family members, guardian, or agent) The contract or annex should be updated when relevant regulations, service recipients, or institutional service conditions change.
	C2: Fees and receipts	The fee standards are subject to approval by the competent authority with regulations.
	C3: Establishment and handling of the feedback/complaint process	Establish procedures and processes for clients/family members to provide feedback and file complaints and ensure that clients/family members are clearly informed of the complaint channels. Regularly analyze feedback and complaints, implement improvement measures accordingly, and ensure proper execution, while keeping records of meetings.
	C4: Service satisfaction surveys	Establish the implementation guidelines for the satisfaction survey (including implementation method, survey timing, and the method for selecting survey cases). Conduct a satisfaction survey at least once a year. Analyze and review according to the survey results and propose improvement measures in quality-related meetings.

Source from: [9,17].

**Table 3:** Two-dimensional gap matrix in four quadrants.

Quadrant	I	II	III	IV
Evaluators (External Committee Evaluation)/Evaluatees (Internal Self-Assessment)	+,+	-,+	-,-	+,-
Definition	Consensus Excellence	Critical Improvement/Overestimation	Consensus Weakness	Potential Advantage/Underestimation
Status Description	Both the evaluators’ and evaluatees’ scores were high. This indicates a strong alignment between actual performance and internal perception.	The evaluators provided lower ratings than the evaluatees’ scores. This indicates a significant perceptual gap where lefts may be overestimating their performance.	Both the evaluators’ and evaluatees’ evaluations yielded low scores. This suggests that service quality deficiencies are evident and recognized by both parties, requiring systemic intervention.	The evaluators‘ evaluation was higher than that of the evaluatees. This indicates that the lefts’ actual performance exceeds their own perceptions, revealing untapped strengths.

Note: “+” indicates scores above the mean; “-” indicates scores below the mean.

**Table 5:** Descriptive statistics of home-based LTC center consensus benchmarks from Evaluatees and Evaluators.

Concept	Group	n	M	SD	Welch’s *t*	df	*p*	Cohen’s d
Management effectiveness	Evaluates	50	4.56	0.31	6.89	47.2	<0.001	1.63
	Evaluators	28	3.95	0.43				
Professional care quality	Evaluates	50	4.52	0.36	6.28	44.8	<0.001	1.57
	Evaluators	28	3.79	0.55				
Individual equity guarantee	Evaluates	50	4.70	0.31	5.55	48.9	<0.001	1.32
	Evaluators	28	4.22	0.40				
Total	Evaluates	50	4.59	0.31	7.21	49.5	<0.001	1.71
	Evaluators	28	3.99	0.39				

n = Sample size; M = Mean; SD = Standard Deviation; df = Degrees of freedom. Cohen’s d thresholds: 0.2 (small), 0.5 (medium), 0.8 (large).

**Table 6:** Difference Analysis of Consensus Benchmark between Evaluatees and Evaluators.

Concept	Consensus Benchmark	Evaluates	Evaluators	M.D.	*t*	*p*	Cohen’s d	Interpretation
A: Management effectiveness	A1	4.505	3.855	0.650	6.177	<0.001 ***	1.48	Extreme Gap
	A2	4.685	4.02	0.665	7.995	<0.001 ***	1.88	Critical Gap
	A3	4.605	3.965	0.640	6.694	<0.001 ***	1.58	Extreme Gap
	A4	4.805	4.455	0.350	4.028	<0.001 ***	0.98	Large Gap
	A5	4.835	4.73	0.105	1.632	0.109	0.38	Consensus
	A6	3.845	3.47	0.375	3.352	0.002	0.82	Large Gap
	A7	4.55	3.94	0.610	6.276	<0.001 ***	1.48	Extreme Gap
	A8	4.61	3.395	1.215	6.567	<0.001 ***	1.54	Max Discrepancy
	A9	4.58	3.675	0.905	6.679	<0.001 ***	1.63	Extreme Gap
	A10	4.505	3.84	0.665	5.001	<0.001 ***	1.25	Extreme Gap
	A11	4.58	4.13	0.450	4.041	<0.001 ***	0.98	Large Gap
B: Professional care quality	B1	4.445	4.015	0.430	4.256	<0.001 ***	0.94	Large Gap
	B2	4.625	4.24	0.385	3.031	0.004	0.76	Moderate-Large
	B3	4.38	3.455	0.925	5.985	<0.001 ***	1.49	Extreme Gap
	B4	4.595	3.695	0.900	7.311	<0.001 ***	1.72	Extreme Gap
	B5	4.565	3.53	1.035	6.831	<0.001 ***	1.61	Extreme Gap
C: Individual equity guarantee	C1	4.805	4.225	0.580	5.838	<0.001 ***	1.44	Extreme Gap
	C2	4.835	4.59	0.245	2.796	0.007	0.66	Moderate Gap
	C3	4.565	3.93	0.635	7.09	<0.001 ***	1.67	Extreme Gap
	C4	4.58	4.145	0.435	5.838	<0.001 ***	1.34	Extreme Gap
Total		4.591	3.987	0.604			1.71	Systemic Gap

*** *p* < 0.001.

**Table 7:** Consensus benchmarks of home-based LTC centers in different quadrants.

Quadrant	Perceptual Nature	Strategic Goal	Key Benchmarks
I: Consensus Excellence	Consensus Strength	Maintain Quality	A2, A3, A4, A5 (True Consensus)
II: Critical Improvement/Overestimation	Critical Blind Spot	Immediate Intervention	A8 ***, A9 ***, B4 ***
III: Consensus Weakness	Consensus Deficiency	Strategic Improvement	A1 ***, A6, B5 ***
IV: Potential Advantages/Underestimation	Hidden Advantage	Recognition & Leverage	B1 ***

*** *p* < 0.001.

**Table 8:** Evidence-Based Strategic Recommendations.

Empirical Evidence (The “What”)	Strategic Recommendation (The “How”)	Policy/Organizational Goal
Evidence A: Significant gaps in Quadrant II (Blind Spots) regarding staff training and case tracking (*p* < 0.001).	Implementation of Digital Early Warning Systems: Transition from manual logs to automated tracking for compliance and personnel updates.	Operational Efficiency: Minimizing human error during staff turnover.
Evidence B: External evaluators prioritize “Emergency Resilience” (B5) over “Daily Stability.”	From SOPs to Dynamic Drills: Shift from static document reviews to active crisis-simulation and coaching models.	Organizational Resilience: Enhancing the capacity to respond to high-risk contingencies.
Evidence C: Managers focus on system integrity, while evaluators focus on “Grievance Responsiveness.”	Standardization vs. Flexibility Balance: Refine indicators to include “Response Speed” and “User Satisfaction” as weighted benchmarks.	Service Equity: Ensuring that formal systems provide functional efficacy for recipients.
Evidence D: Quadrant IV (Hidden Strengths) shows staff competence exceeds manager awareness.	Professional Branding & Recognition: Formalize internal recognition of expertise to leverage latent organizational assets.	Competitive Advantage: Improving institutional reputation in the LTC market.

## Data Availability

The data presented in this study are available on request from the corresponding author due to confidentiality considerations and institutional data protection regulations.

## References

[1] Ministry of Health and Welfare (MOHW) (2023). Long-Term Care 10-Year Plan 2.0: Long-Term Care Service Quality Verification Mechanism. https://1966.gov.tw/LTC/lp-6485-207.html.

[2] National Development Council (NDC) (2024). Taiwan Demographic Data Report. https://pop-proj.ndc.gov.tw/News.aspx?n=3&sms=10347.

[3] Yisel M.G.L.l., Simón Q.R.L. (2025). Assessment of Patients’ Quality of Care in Healthcare Systems: A Comprehensive Narrative Literature Review. Healthcare.

[4] Audit Department (2023). Report on the Implementation of the Government’s 10-Year Plan for Long-Term Care 2.0. https://www.audit.gov.tw/p/405-1000-8867,c158.php?Lang=zh-tw.

[5] Ting T.-H. (2023). Preliminary Study on the Quality of Care in Home Care Services. Master’s Thesis.

[6] National Development Council (NDC) (2024). Population Projections. http://csyue.nccu.edu.tw/ch/Taiwan%20Population%20Projection%20(2024-2070).pdf.

[7] Wu S.-C., Chu F.-H., Lin L.-C., Chen S.-H., Chang M.-M., Chou L.-H., Ye J.-L., Yang Y.-R. (2019). To establish the accreditation indices for the community and home-based long-term care facilities of Taipei City. J. Long-Term Care.

[8] Liao C.-C., Chang S.-C., Hung C.-T., Chen N.-S., Hwu Y.-J. (2022). Results and related factors of integrated community service center accreditation. Hospital.

[9] Hung J.-Y. (2024). 2024 Yunlin County Long-Term Care Service Quality Improvement Plan Achievement Report.

[10] Yalda M., Hessane H., Azizollah A., Reza E., Mohammad M. (2026). Evaluation of the hospital service quality using the Importance–Performance Analysis (IPA) tool in Ardabil city. J. Educ. Health Promot..

[11] Francisco J.M., Antonio C., Luis R.M., Juan V. (2010). An Importance-Performance Analysis of Primary Health Care Services: Managers vs. Patients Perceptions. J. Serv. Sci. Manag..

[12] Chen C.-W., Pai J.-Y., Zeng S.-H., Chen W.-C. (2016). A study of service quality on the long-term care institution residents’ satisfaction and reuse intention. J. Health Manag..

[13] Caughey G.-E., Rahja M., Harrison S., Fernando R., Inacio M.-C. (2025). Quality indicators to monitor home care services for the older population: A scoping review. J. Am. Med. Dir. Assoc..

[14] Zheng Q.-L., Kong L.-N., Hu P., Liu D.-X. (2024). Identifying quality indicators for home care services: A modified Delphi and analytic hierarchy process study. BMC Nurs..

[15] Wang H., Coyte P.C., Shi W., Zong X., Zhong R. (2025). Social Governance and Sustainable Development in Elderly Services: Innovative Models, Strategies, and Stakeholder Perspectives. Sustainability.

[16] Gao Y., Yang Y., Qin T., Li X., Guo J., Zhou L., Gu M., Wang Y. (2025). Improving Service Quality of Home-Based Health Care Services for the Elderly: A Qualitative Study of Facilitators and Barriers Reported by Community Health Workers in Beijing. J. Multidiscip. Healthc. PMC Qual. Study.

[17] Hung J.-Y. (2026). Assessment of Home- and Community-Based Long-Term Care Centers in Taiwan. Soc. Sci..

[18] Martilla J.-A., James J.-C. (1977). Importance-performance analysis. J. Mark..

[19] Chen L.-J., Wang J.-H., Chen M.-T. (2019). Opinion difference between evaluators and providers on home care service assessment system. J. Healthc. Manag..

[20] Cheng C.-Y. (2025). Applying IPA model to analyze the care quality of home care attendants. J. Geotechnol. Serv. Manag..

[21] Chen C.-S. (2024). The Impact of Long-Term Care Service Evaluation on the Management of Home-Based Long-Term Care Institutions: A Case Study of Taichung City. Master’s Thesis.

